# Anemia in hospitalized patients with pulmonary tuberculosis*

**DOI:** 10.1590/S1806-37132014000400008

**Published:** 2014

**Authors:** Marina Gribel Oliveira, Karina Neves Delogo, Hedi Marinho de Melo Gomes de Oliveira, Antonio Ruffino-Netto, Afranio Lineu Kritski, Martha Maria Oliveira

**Affiliations:** Academic Program in Tuberculosis, Federal University of Rio de Janeiro School of Medicine, Rio de Janeiro, Brazil; Academic Program in Tuberculosis, Federal University of Rio de Janeiro School of Medicine, Rio de Janeiro, Brazil; Hospital Estadual Santa Maria, Rio de Janeiro State Department of Health, Rio de Janeiro, Brazil; University of São Paulo at Ribeirão Preto School of Medicine, Ribeirão Preto, Brazil; Federal University of Rio de Janeiro School of Medicine, Rio de Janeiro, Brazil; Academic Program in Tuberculosis, Federal University of Rio de Janeiro School of Medicine, Rio de Janeiro, Brazil

**Keywords:** Tuberculosis, pulmonary, Anemia, Malnutrition, Iron

## Abstract

**OBJECTIVE:**

To describe the prevalence of anemia and of its types in hospitalized patients with pulmonary tuberculosis.

**METHODS:**

This was a descriptive, longitudinal study involving pulmonary tuberculosis inpatients at one of two tuberculosis referral hospitals in the city of Rio de Janeiro, Brazil. We evaluated body mass index (BMI), triceps skinfold thickness (TST), arm muscle area (AMA), ESR, mean corpuscular volume, and red blood cell distribution width (RDW), as well as the levels of C-reactive protein, hemoglobin, transferrin, and ferritin.

**RESULTS:**

We included 166 patients, 126 (75.9%) of whom were male. The mean age was 39.0 ± 10.7 years. Not all data were available for all patients: 18.7% were HIV positive; 64.7% were alcoholic; the prevalences of anemia of chronic disease and iron deficiency anemia were, respectively, 75.9% and 2.4%; and 68.7% had low body weight (mean BMI = 18.21 kg/m^2)^. On the basis of TST and AMA, 126 (78.7%) of 160 patients and 138 (87.9%) of 157 patients, respectively, were considered malnourished. Anemia was found to be associated with the following: male gender (p = 0.03); low weight (p = 0.0004); low mean corpuscular volume (p = 0.03);high RDW (p = 0; 0003); high ferritin (p = 0.0005); and high ESR (p = 0.004). We also found significant differences between anemic and non-anemic patients in terms of BMI (p = 0.04), DCT (p = 0.003), and ESR (p < 0.001).

**CONCLUSIONS:**

In this sample, high proportions of pulmonary tuberculosis patients were classified as underweight and malnourished, and there was a high prevalence of anemia of chronic disease. In addition, anemia was associated with high ESR and malnutrition.

## Introduction

According to the World Health Organization, one third of the world population is infected with *Mycobacterium tuberculosis*. It is estimated that approximately 8.8 million new cases of tuberculosis occur each year; Brazil ranks 18th among the 22 countries that collectively account for most such cases.^(^ 1 ^)^

In Brazil, approximately 85,000 cases of tuberculosis occur each year, approximately 5,000 deaths being associated with the disease. The incidence rate of tuberculosis in the country has been estimated at 37.2/100,000 population. Among all Brazilian states, Rio de Janeiro has the highest annual incidence rate of tuberculosis (73.27/100,000 population) and the highest mortality rate (5.0/100,000 population).^(^ 2 ^)^

According to the World Health Organization, the severity of the global tuberculosis situation is primarily due to social inequality, population aging, large migration flows, and the advent of AIDS in the 1980s.^(^ 3 ^)^ In addition to AIDS, risk factors for tuberculosis include alcoholism, smoking, history of tuberculosis, diabetes mellitus, malnutrition, and low socioeconomic status.^(^ 4 ^)^

The association between tuberculosis and malnutrition consists of two interactions: the effect of tuberculosis on the nutritional status and the effect of malnutrition on the clinical manifestations of tuberculosis, as a result of immunological impairment.^(^ 3 ^,^ 5 ^)^ Anemia has been observed in 32-94% of patients with tuberculosis ^(^ 6 ^-^ 8 ^)^

Iron deficiency is the most common micronutrient deficiency in the world, and numerous studies have evaluated the association between serum iron levels and iron-deficiency anemia.^(^ 9 ^,^ 10 ^)^ However, there is controversy regarding the administration of iron; some studies have shown that iron deficiency increases susceptibility to infectious processes, whereas others have shown that excess iron is more harmful to the human body than is iron deficiency, and that iron deficiency can protect against infection.^(^ 11 ^)^

Among the anemias that are characterized by altered iron metabolism, iron-deficiency anemia and anemia of chronic disease are the most common.^(^ 12 ^)^

Iron-deficiency anemia is the most common nutritional deficiency worldwide, affecting primarily individuals residing in developing countries. It occurs as a result of chronic blood loss, urinary losses, poor iron intake/absorption, and increased blood volume. In individuals with iron-deficiency anemia, a decrease in plasma iron levels occurs, limiting erythropoiesis. The risk of developing iron-deficiency anemia is highest among infants, children under 5 years of age, and women of childbearing age.^(^ 12 ^)^

Anemia of chronic disease, also known as anemia of inflammation, is a clinical syndrome characterized by the development of anemia in patients with (fungal, bacterial, or viral) infectious diseases, such as tuberculosis, inflammatory diseases, autoimmune diseases, and neoplastic diseases.^(^ 13 ^)^ It is characterized by mild to moderate normocytic hypochromic anemia, and hypochromia and microcytosis can occur in 20-30% of cases. However, when microcytosis occurs, it is not as pronounced as it is in iron-deficiency anemia.^(^ 12 ^)^ This type of anemia is associated with decreased serum iron levels and total iron binding capacity, as well as with increased ferritin levels.^(^ 13 ^)^

In patients with active tuberculosis, few of the studies investigating the presence of anemia have determined whether anemia is associated with iron deficiency or chronic disease or have identified variables associated with its occurrence.^(^ 6 ^-^ 8 ^)^

The objective of the present study was to describe the prevalence of anemia and of its types in hospitalized patients with pulmonary tuberculosis, as well as to examine the relationship between anemia and the clinical and nutritional status of anemic patients in comparison with non-anemic patients. 

## Methods

This was a prospective cross-sectional descriptive study, which included active pulmonary tuberculosis patients consecutively admitted to one of two tuberculosis referral hospitals in the state of Rio de Janeiro (namely *Instituto Estadual de Doenças do Tórax Ary Parreiras* and *Hospital Estadual Santa Maria*, both located in the city of Rio de Janeiro) and initiating antituberculosis treatment between March of 2007 and December of 2010. All participants gave written informed consent. 

Patients under 18 years of age or over 60 years of age were excluded, as were those who had previously undergone tuberculosis treatment or who had been receiving treatment with antituberculosis drugs for more than seven days; those with diabetes mellitus receiving insulin therapy; those with renal failure on peritoneal dialysis or hemodialysis; those who had received blood transfusions in the 3 months preceding study entry; and those who were pregnant or lactating. For data collection, we used a standardized questionnaire and reviewed medical records. In addition, we collected blood samples and performed medical and nutritional assessment up to seven days after the initiation of pharmacological treatment. Alcohol abuse was defined as a daily intake of 30 g or more for males and of 24 g or more for females. The Cut down, Annoyed, Guilty, and Eye-opener (CAGE) questionnaire was used in order to identify alcohol abuse.^(^ 14 ^)^

Nutritional assessment included measurements of weight, height, and body mass index (BMI), in order to identify patients who were underweight,^(^ 15 ^)^ as well as measurements of triceps skinfold thickness (TST) and arm muscle area (AMA), in order to identify patients who were malnourished. ^(^ 15 ^,^ 16 ^)^

**Chart 1:**
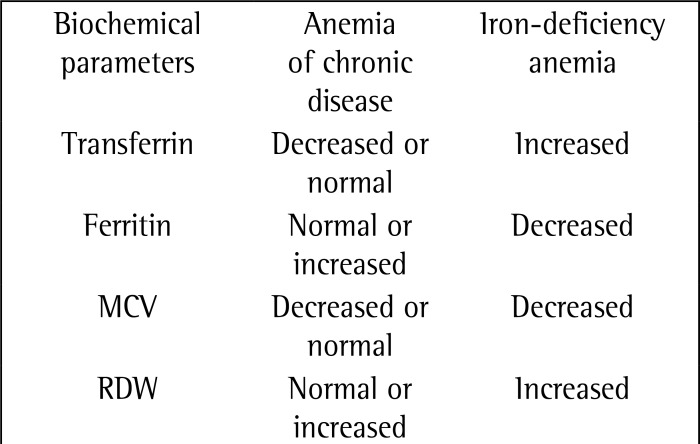
Parameters for the evaluation of the types of anemia studied. MCV: mean corpuscular volume; and RDW: red blood cell distribution width

In order to classify anemia, we analyzed the following parameters: hemoglobin levels; transferrin levels; ferritin levels; and mean corpuscular volume (MCV). We used red blood cell distribution width (RDW) in order to assess the presence of anisocytosis. This classification is shown in Chart 1. In addition to the aforementioned measurements, we performed measurements of C-reactive protein (CRP) and ESR, as well as HIV testing. All tests were performed in a laboratory certified by the Brazilian Clinical Pathology Association Clinical Laboratory Accreditation Program. Iron-deficiency anemia was characterized by decreased levels of iron and ferritin and increased levels of transferrin, whereas anemia of chronic disease was characterized by decreased levels of iron and transferrin and increased levels of ferritin.^(^ 13 ^)^

For statistical analysis, we used descriptive statistics, including range (minimum and maximum values), mean, standard deviation, median, interquartile range, and 95% CI. We used the Kolmogorov-Smirnov test in order to test the normality of the variables and Levene's test in order to determine the equality of variances. We used the Student's t-test in order to compare means with normal distribution between the groups of patients with and without anemia. We used ANOVA in order to analyze the differences among quantitative variables and the chi-square test in order to identify associations among categorical variables. For the identification of variables associated with anemia, we used multivariate logistic regression analysis in order to assess the presence of confounding covariates. Covariates with values of p < 0.20 in the bivariate analysis were included in the model. Values of p < 0.05 were considered statistically significant. All analyses were performed with the Statistical Package for the Social Sciences, version 16.0 for Windows (SPSS Inc., Chicago, IL, USA). 

The present study was approved by the Research Ethics Committee of the Federal University of Rio de Janeiro School of Medicine Clementino Fraga Filho University Hospital on April 28, 2005 (Protocol no. 004/05). 

## Results

We included 166 patients, 126 (75.9%) of whom were male. The mean age was 39.0 ± 10.7 years. In our sample, 95 (62.5%) of 152 patients were non-White; 18 (18.7%) of 96 patients were HIV-positive; 97 (64.7%) of 150 patients were considered alcoholic on the basis of the CAGE questionnaire; 118 (74.7%) of 158 patients were classified as smokers or former smokers; and 47 (30.1%) of 156 patients reported illicit drug use. Of the 166 patients, 18 (10.9%) had no anemia and 148 (89.1%) had anemia. Of those, 4 (2.4%) had iron-deficiency anemia and 126 (75.9%) had anemia of chronic disease; in the remaining 18 patients, it was impossible to distinguish between the two. 

We found low hemoglobin levels (mean, 10.86 ± 2.04 g/dL) in 89.2% of patients; low transferrin levels (mean, 177.28 ± 58.71 mg/dL) in 65.3%; and low MCV (mean, 82.00 ± 7.77 fL) in 39.7%. In addition, we found high ferritin levels (mean, 520.68 ± 284.26 ng/mL) in 52.7% of patients; high RDW (mean, 16.36 ± 3.47%) in 55.4%; high CRP levels (mean, 5.84 ± 4.22 mg/dL) in 98.2%; and high ESR (mean, 60.30 ± 39.84 mm/h) in 84.3%. 

On the basis of the BMI, 88 (68.7%) of 128 patients were underweight (mean, 18.21 ± 2.93 kg/m^2)^. On the basis of the TST, 126 (78.7%) of 160 patients were mildly, moderately, or severely malnourished (mean, 6.16 ± 3.83 mm). On the basis of the AMA, 138 (87.9%) of 157 patients were mildly, moderately, or severely malnourished (mean, 24.41 ± 9.86 cm^2)^. 

When we compared the sociodemographic and clinical variables between the groups of patients with and without anemia, we found an association of anemia with the male gender (p = 0.03) and a trend toward an association of anemia with being a smoker or former smoker (p = 0.05; Tables 1 and 2). Table 3 shows a comparison of nutritional and laboratory variables between the groups of patients with and without anemia. Anemia was found to be associated with the following: BMI (p = 0.0004); MCV (p = 0.03); ferritin (p = 0.0005); RDW (p = 0.0003); and ESR (p = 0.004). After the multivariate analysis, ESR was the only independent variable that remained. 

**Table 1:** Distribution of sociodemographic variables between the groups of patients with and without anemia.a

Variable	Patients with anemia	Patients without anemia	OR (95% CI)	p
	(n = 148)	(n = 18)		
Gender				
Male	116 (78.4)	10 (55.6)	2.90 (1.05-7.95)	0.03
Female	32 (21.6)	8 (44.4)		
Age, years^b^	38.6	37.6		0.71
Smoking status				
Smokers	70 (47.3)	9 (50.0)	2.70 (0.98-7.41)	0.05
Former smokers	38 (25.7)	1 (5.6)		
Never smokers	32 (21.6)	8 (44.4)		
ND	8 (5.4)	0 (0.0)		
Illicit drug use				
Yes	43 (29.1)	4 (22.2)	1.58 (0.49-5.09)	0.43
No	95 (64.2)	14 (77.8)		
ND	10 (6.8)	0 (0.0)		

ND : no data

a Values expressed as n (%), except where otherwise indicated

b Values expressed as mean.

**Table 2:** Distribution of clinical variables between the groups of patients with and without anemia.a

Variable	Patients with anemia	Patients without anemia	OR (95% CI)	p
	(n = 148)	(n = 18)		
HIV status				
Positive	17 (11.5)	1 (5.6)	2.79 (0.33-23.1)	0.32
Negative	67 (45.3)	11 (61.1)		
ND	64 (43.2)	6 (33.3)		
Alcoholism				
Yes	90 (60.8)	7 (38.9)	2.28 (0.77-6.70)	0.12
No	45 (30.4)	8 (44.4)		
ND	13 (8.8)	3 (16.7)		
Transferrin levels				
Low	86 (58.1)	10 (55.6)	1.36 (0.48-3.83)	0.55
Normal	44 (29.7)	7 (38.9)		
ND	18 (12.2)	1 (5.8)		
Ferritin levels				
High	77 (52)	1 (5.6)	24.6 (3.2-191.2)	0.00005
Normal	49 (33.1)	10 (55.6)		
Low	4 (2.7)	7 (38.9)		
ND	18 (12.2)	0 (0.0)		
MCV				
Low	63 (42.6)	3 (16.7)	3.70 (1.02-13.35)	0.03
Normal	85 (57.4)	15 (83.3)		
RDW				
Low	4 (2.7)	12 (66.7)	0.03 (0.004-0.26)	0.0003
Normal	52 (35.1)	6 (33.3)		
High	92 (62.2)	0 (0.0)		
CRP levels				
Normal	2 (1.3)	1 (0.6)	0.23 (0.02-2.72)	0.21
High	145 (98.0)	17 (94.4)		
ND	1 (0.7)	0 (0.0)		
ESR				
Normal	19 (12.8)	7 (38.9)	0.23 (0.07-0.67)	0.004
High	129 (87.2)	11 (61.1)		

ND : no data

MCV : mean corpuscular volume

RDW : red blood cell distribution width

CRP : C-reactive protein

a Values expressed as n (%).

**Table 3:** Distribution of anthropometric variables between the groups of patients with and without anemia.a

Variable	Patients with anemia	Patients without anemia	OR (95% CI)	p
	(n = 148)	(n = 18)		
BMI				
Underweight	82 (55.4)	6 (33.3)	5.85 (2.00-17.07)	0.0004
Normal weight	25 (31.1)	11 (61.1)		
Overweight	3 (2.0)	1 (5.6)		
ND	17(11.5)	0 (0.0)		
Nutritional status (TST)				
Severe malnutrition	85 (57.4)	9 (50)	2.24 (0.76-6.57)	0.13
Mild/moderate malnutrition	30 (20.3)	2 (11.1)		
Normal nutritional status	28 (18.9)	6 (33.3)		
ND	5 (3.4)	1 (5.6)		
Nutritional status (AMA)				
Severe malnutrition	117 (79.1)	11 (61.1)	2.56 (0.74-8.87)	0.12
Mild/moderate malnutrition	8 (5.4)	2 (11.1)		
Normal nutritional status	15 (10.1)	4 (22.2)		
ND	8 (5.4)	1 (5.6)		

BMI : body mass index

ND : no data

TST : triceps skinfold thickness

AMA : arm muscle area

a Values expressed as n (%).

Table 4 shows the results of the correlation of nutritional and laboratory variables with the presence of anemia. Mean BMI and mean TST were significantly lower in the patients with anemia than in those without. However, high ESR values were significantly associated with anemia (p < 0.001). Nevertheless, there were no significant differences between the groups of patients with and without anemia regarding AMA, transferrin levels, ferritin levels, or MCV. 

**Table 4:** Correlation between nutritional and laboratory variables in the groups of patients with and without anemia.

Variable	Patients with anemia		Patients without anemia		p*
	n	Mean ± SD	n	Mean ± SD	
BMI	131	18.047 ± 2.85	17	19.55 ± 3.22	0.044
TST	143	5.85 ± 3.44	17	8.7 ± 5.72	0.003
AMA	140	24.12 ± 9.95	17	26.88 ± 8.92	0.276
Transferrin	130	175.71 ± 59.64	17	189.29 ± 51.06	0.372
Ferritin	130	534.30 ± 266.98	17	416.54 ± 313.34	0.403
MCV	148	81.83 ± 7.99	18	83.42 ± 5.66	0.415
RDW	148	16.54 ± 3.50	18	14.94 ± 2.92	0.065
CRP	147	6.06 ± 4.23	18	4.04 ± 3.89	0.055
ESR	148	69.49 ± 39.56	18	25.89 ± 21.56	< 0.001

BMI : body mass index

TST : triceps skinfold thickness

AMA : arm muscle area

MCV : mean corpuscular volume

RDW : red blood cell distribution width

CRP : C-reactive protein

* Student's t-test.

## Discussion

In the present study, pulmonary tuberculosis was found to be more common in young adults, males, alcoholics, smokers, illicit drug users, and HIV-positive patients; this finding is similar to those reported in studies evaluating pulmonary tuberculosis inpatients at general and tuberculosis referral hospitals in Brazil.^(^ 17 ^,^ 18 ^)^

The prevalence of anemia in the present study (89.2%) was higher than was that in a study conducted in South Korea (32%)^(^ 6 ^)^ and similar to that in studies conducted in Indonesia (63%),^(^ 7 ^)^ Tanzania (96%),^(^ 8 ^)^ and Malawi (88%).^(^ 19 ^)^ In the present study, the proportion of patients with anemia of chronic disease was higher than was that of those with iron-deficiency anemia (75.9% vs. 2.4%), a finding that was similar to those reported in other studies^(^ 6 ^,^ 7 ^)^ but different from those reported in another study.^(^ 8 ^)^ In the bivariate analysis, anemia was found to be more common in males than in females, a finding that is inconsistent with the literature.^(^ 6 ^-^ 8 ^)^ However, the association between anemia and the male gender was not confirmed in the multivariate analysis; likewise, we found no association between anemia and HIV infection, a finding that is in disagreement with those reported in other studies.^(^ 8 ^,^ 19 ^)^

On the basis of the BMI, 68.7% of patients were found to be underweight, a proportion that is higher than that reported in a study conducted in Peru (21%)^(^ 20 ^)^ and similar to those reported in studies conducted in Malawi^(^ 4 ^,^ 21 ^)^ and England.^(^ 22 ^)^ This is probably due to the fact that those studies included high proportions of HIV-positive inpatients. 

On the basis of the TST and AMA, 126 (78.7%) of 160 patients and 138 (87.9%) of 157 patients, respectively, were considered malnourished. Similar results have been reported elsewhere.^(^ 22 ^)^ A 13% reduction in TST and a 20% reduction in AMA were reported in a case-control study,^(^ 22 ^)^ whereas a 35% reduction in TST and a 19% reduction in AMA were reported in another study.^(^ 21 ^)^

Almost all of the patients included in our study were found to have elevated levels of CRP and ESR, a finding that is similar to those reported in the literature.^(^ 7 ^,^ 23 ^,^ 24 ^)^ We believe that CRP and ESR can be useful as markers of the effect of treatment and of the resolution of inflammation, given that CRP and ESR levels decreased during antituberculosis treatment, having normalized by the end of the treatment period (data not shown). 

The concentrations of most proteins are elevated in tuberculosis patients, the exception being the concentrations of transferrin and hemoglobin, which are decreased.^(^ 25 ^)^ In our study, we found low concentrations of transferrin and high concentrations of ferritin, a finding that is similar to those reported by other groups of authors.^(^ 6 ^,^ 7 ^,^ 25 ^,^ 26 ^)^

In conditions other than inflammatory conditions, determination of ferritin levels is the most sensitive method for the diagnosis of iron deficiency. However, in tuberculosis patients, determination of ferritin levels should be used with caution because ferritin levels do not accurately express the amount of iron in such patients. Therefore, patients can have iron deficiency even when they have normal or increased ferritin levels.^(^ 13 ^)^

Given that microcytosis was observed in most of the patients in the present study, increased RDW might be useful to demonstrate iron deficiency,^(^ 26 ^)^ although its role remains controversial.^(^ 27 ^)^

When we compared the groups of patients with and without anemia in terms of their nutritional status, we found that malnutrition was more severe in the former, who had low serum concentrations of transferrin and high serum concentrations of ferritin, as reported in one study.^(^ 7 ^)^ Regarding the inflammatory state, the multivariate analysis showed that ESR was higher in the patients with anemia than in those without, the difference being significant. One group of authors^(^ 27 ^)^ found that ESR increases in response to anemia, a finding that corroborates the results of the present study. However, although we excluded patients with a history of tuberculosis, those receiving insulin therapy, those on peritoneal dialysis or hemodialysis, and those who had received blood transfusions in the 3 months preceding study entry, the associations of ESR and CRP with anemia in the present study should be confirmed in studies investigating larger samples, preferably with a higher prevalence of iron-deficiency anemia and without the presence of comorbidities such as HIV infection, alcoholism, and smoking. 

Given that it was impossible to use all of the recommended parameters for the differential diagnosis between iron-deficiency anemia and anemia of chronic disease, including transferrin receptor and bone marrow analysis,^(^ 13 ^)^ the criteria used in the present study resulted in a low frequency of iron-deficiency anemia in isolation. However, we believe that some of the patients with anemia of chronic disease also had iron-deficiency anemia, as reported in one study.^(^ 8 ^)^ In such cases, not all patients benefit from iron supplementation.^(^ 13 ^)^ In another study,^(^ 7 ^)^ after successful tuberculosis treatment, anemia was corrected without iron supplementation in most patients. 

In conclusion, high proportions of pulmonary tuberculosis patients were classified as underweight and malnourished on the basis of different parameters (BMI, AMA, and TST), and there was a high prevalence of anemia of chronic disease. In addition, the degree of malnutrition was higher in the patients with anemia than in those without. 
