# Supplementary material for: Anemia in hospitalized patients with pulmonary tuberculosis
Source: J Bras Pneumol. 2014 Jul-Aug;40(4):403–10. doi: 10.1590/S1806-37132014000400008 (PMC4201171; doi:10.1590/S1806-37132014000400008)
Supplement: Translation

# Translation: Anemia em pacientes internados com tuberculose pulmonar*

**Authors:** Marina Gribel Oliveira, Karina Neves Delogo, Hedi Marinho de Melo Gomes de Oliveira, Antonio Ruffino-Netto, Afranio Lineu Kritski, Martha Maria Oliveira

**Affiliations:** Programa Acadêmico de Tuberculose, Faculdade de Medicina, Universidade Federal do Rio de Janeiro, Rio de Janeiro (RJ) Brasil; Programa Acadêmico de Tuberculose, Faculdade de Medicina, Universidade Federal do Rio de Janeiro, Rio de Janeiro (RJ) Brasil; Hospital Estadual Santa Maria, Secretaria Estadual de Saúde do Rio de Janeiro, Rio de Janeiro (RJ) Brasil; Faculdade de Medicina de Ribeirão Preto, Universidade de São Paulo, Ribeirão Preto (SP) Brasil; Faculdade de Medicina, Universidade Federal do Rio de Janeiro, Rio de Janeiro (RJ) Brasil; Programa Acadêmico de Tuberculose, Faculdade de Medicina, Universidade Federal do Rio de Janeiro, Rio de Janeiro (RJ) Brasil

**Keywords:** Tuberculose pulmonar, Anemia, Desnutrição, Ferro

## Abstract

**OBJETIVO:**

Descrever a prevalência de anemia e de seus tipos em pacientes internados com tuberculose pulmonar.

**MÉTODOS:**

Estudo descritivo e longitudinal com pacientes com tuberculose pulmonar hospitalizados em dois hospitais de referência na cidade do Rio de Janeiro (RJ). Foram avaliados o índice de massa corpórea (IMC), dobra cutânea tricipital (DCT), área muscular do braço (AMB), VHS, volume globular médio e *red blood cell distribution width* (RDW, índice de anisocitose eritrocitária), assim como os níveis de proteína C reativa, hemoglobina, transferrina e ferritina.

**RESULTADOS:**

Foram incluídos 166 pacientes, sendo 126 (75,9%) do sexo masculino. A média de idade foi de 39,0 ± 10,7 anos. Alguns dados não estavam disponíveis para todos os pacientes: 18,7% eram portadores de HIV; 64,7% eram etilistas; as prevalências de anemia da doença crônica e de anemia ferropriva foram, respectivamente, de 75,9% e 2,4%; e 68,7% apresentaram baixo peso (média do IMC = 18,21 kg/m^2)^. Com base em DCT e AMB, respectivamente, 126/160 pacientes (78,7%) e 138/157 pacientes (87,9%) foram considerados desnutridos. A presença de anemia associou-se às seguintes variáveis: sexo masculino (p = 0,03), baixo peso (p = 0,0004), baixo volume globular médio (p = 0,03), alto RDW (p = 0,0003), alto nível de ferritina (p = 0,0005) e de VHS (p = 0,004). Houve diferenças significativas entre pacientes anêmicos e não anêmicos em relação a IMC (p = 0,04), DCT (p = 0,003) e VHS (p < 0,001).

**CONCLUSÕES:**

Nesta amostra, a proporção de pacientes com tuberculose pulmonar classificados com baixo peso e desnutrição foi elevada, assim como a prevalência de anemia da doença crônica. Além disso, a anemia associou-se a VHS elevada e desnutrição.

## Introdução

Segundo a Organização Mundial de Saúde, um terço da população mundial está infectada por My*cobacterium tuberculosis.* O número anual de novos casos de tuberculose é estimado em cerca de 8,8 milhões, e a maioria está concentrada em 22 países, entre os quais o Brasil ocupa o 18º lugar.^(^ 1 ^)^

O Brasil apresenta aproximadamente 85 mil casos por ano e cerca de 5 mil mortes associadas à tuberculose. O coeficiente de incidência de tuberculose para o país foi de 37,2/100.000 habitantes. O estado do Rio de Janeiro tem a maior taxa de incidência anual de tuberculose do país (73,27/100.000 habitantes), assim como a maior taxa de mortalidade (5,0/100.000 habitantes).^(^ 2 ^)^

A Organização Mundial de Saúde assinala como as principais causas para a gravidade da situação mundial da tuberculose a desigualdade social, o envelhecimento da população, os grandes movimentos migratórios e o advento da AIDS a partir da década de 80 do século passado.^(^ 3 ^)^ Além da AIDS, outros fatores de risco para tuberculose são o etilismo, o tabagismo, a história de tuberculose, o diabetes mellitus, a desnutrição e as baixas condições socioeconômicas.^(^ 4 ^)^

A associação entre tuberculose e desnutrição consiste em duas interações: o efeito da tuberculose no estado nutricional e o efeito da desnutrição nas manifestações clínicas da tuberculose, devido ao comprometimento imunológico dos pacientes. ^(^ 3 ^,^ 5 ^)^ A anemia tem sido observada entre 32% e 94% dos pacientes com tuberculose.^(^ 6 ^-^ 8 ^)^

A deficiência de ferro é a mais comum deficiência de micronutrientes no mundo, e inúmeros relatos têm avaliado a associação de níveis séricos de ferro com a presença de anemia ferropriva.^(^ 9 ^,^ 10 ^)^ Entretanto, existem controvérsias sobre a administração de ferro, pois alguns estudos revelam que a carência de ferro aumenta a suscetibilidade aos processos infecciosos, enquanto outros relatam que o excesso de ferro é mais prejudicial ao organismo humano e que a deficiência de ferro teria até um papel protetor contra as infecções.^(^ 11 ^)^

No grupo das anemias caracterizadas por distúrbios do metabolismo do ferro, podemos classificar a anemia ferropriva e a anemia da doença crônica como sendo as mais comuns.^(^ 12 ^)^

A anemia ferropriva representa a deficiência nutricional de maior ocorrência em todo mundo, atingindo principalmente indivíduos que residem em países em desenvolvimento. Ocorre como resultado de perda sanguínea crônica, perdas urinárias, ingestão e/ou absorção deficiente de ferro e aumento do volume sanguíneo. Na anemia ferropriva ocorre a diminuição dos níveis plasmáticos de ferro, limitando a eritropoese. Os grupos mais vulneráveis para o desenvolvimento desse tipo de anemia são lactentes, crianças menores de 5 anos e mulheres em idade fértil.^(^ 12 ^)^

A anemia da doença crônica, também chamada de anemia da inflamação, é uma síndrome clínica que se caracteriza pelo desenvolvimento da anemia em pacientes que apresentam doenças infecciosas (fúngicas, bacterianas ou virais), tais como a tuberculose, doenças inflamatórias, doenças autoimunes e doenças neoplásicas.^(^ 13 ^)^ Caracteriza-se por uma anemia de grau leve a moderado, normocítica e hipocrômica, podendo ainda ocorrer hipocromia e microcitose em 20-30% dos pacientes. No entanto, a microcitose, quando ocorre, não é tão acentuada como na anemia ferropriva.^(^ 12 ^)^ Esse tipo de anemia está associado à diminuição da concentração do ferro sérico e da capacidade total de ligação do ferro, assim como a níveis aumentados de ferritina.^(^ 13 ^)^

Em pacientes com tuberculose ativa, são poucos os estudos que, ao avaliarem a presença de anemia, distinguiram se essa era associada ao déficit de ferro ou à doença crônica ou que tenham identificado variáveis associadas a sua ocorrência.^(^ 6 ^-^ 8 ^)^

O presente estudo visou descrever o tipo de anemia prevalente nos pacientes com tuberculose pulmonar e sua relação com o estado nutricional e clínico desses pacientes em comparação com pacientes não anêmicos.

## Métodos

Trata-se de um estudo descritivo, de corte transversal e prospectivo, que incluiu pacientes com diagnóstico de tuberculose pulmonar ativa internados consecutivamente em um de dois hospitais de referência para tuberculose no estado do Rio de Janeiro (Instituto Estadual de Doenças do Tórax Ary Parreiras e Hospital Estadual Santa Maria, ambos localizados na cidade do Rio de Janeiro) e que iniciaram o esquema antituberculose no período entre março de 2007 e dezembro de 2010. Todos os participantes assinaram o termo de consentimento livre e esclarecido.

Foram excluídos pacientes com idade inferior a 18 anos ou superior a 60 anos, aqueles com tratamento prévio de tuberculose ou em uso de fármacos antituberculose por período superior a sete dias, portadores de diabetes mellitus em insulinoterapia, portadores de insuficiência renal em diálise peritoneal ou hemodiálise, pacientes hemotransfundidos nos 3 meses anteriores à inclusão no estudo e mulheres no período de gestação ou lactação. Para a coleta de dados no grupo de estudo, foram utilizados um questionário padronizado e consulta aos prontuários médicos, assim como foram realizadas coletas de amostras de sangue e avaliação médica e nutricional em até sete dias após o início do tratamento medicamentoso. O uso abusivo de álcool foi caracterizado como a ingestão diária de 30 g ou mais para homens e de 24 g ou mais para mulheres. O questionário CAGE, cujo nome é um acrônimo de suas quatro questões, foi utilizado para a identificação de abuso de álcool.^(^ 14 ^)^

Para a avaliação nutricional, foram realizadas as seguintes aferições: peso, altura e índice de massa corpórea (IMC), para a classificação de baixo peso,^(^ 15 ^)^ assim como dobra cutânea tricipital (DCT) e área muscular do braço (AMB), para a classificação de desnutrição.^(^ 15 ^,^ 16 ^)^

Para a classificação e diferenciação dos tipos de anemia estudados, foram analisados os seguintes parâmetros: hemoglobina, transferrina, ferritina e volume globular médio (VGM). O *red blood cell distribution width* (RDW, índice de anisocitose eritrocitária) foi utilizado para avaliar a presença de anisocitose. Essa classificação pode ser vista no Quadro 1. Ademais, foram realizadas dosagens de proteína C reativa (PCR), VHS e teste anti-HIV. Todos os exames foram realizados em um laboratório certificado pelo Programa de Acreditação de Laboratórios Clínicos da Sociedade Brasileira de Patologia Clínica. A anemia ferropriva foi caracterizada por níveis reduzidos de ferro e de ferritina e níveis aumentados de transferrina, enquanto a anemia da doença crônica foi caracterizada por níveis reduzidos de ferro e transferrina e níveis aumentados de ferritina.^(^ 13 ^)^

**Quadro 1:** Parâmetros para a avaliação dos tipos de anemia estudados. VGM: volume globular médio; e RDW: red blood cell distribution width (índice de anisocitose eritrocitária).

Para a análise estatística, utilizaram-se estatísticas descritivas, tais como amplitude de variação (valor mínimo e máximo), média, desvio-padrão, mediana, intervalo interquartílico e IC95%. Foram utilizados o teste de Kolmogorov-Smirnov para a verificação da normalidade das variáveis e o teste de Levene para a comparação de igualdade das variâncias. O teste t de Student foi utilizado para comparar médias com distribuição normal entre os pacientes com e sem anemia. A ANOVA também foi utilizada para estimar diferenças entre as variáveis quantitativas e foi utilizado o teste de qui-quadrado para avaliar a associação entre variáveis categóricas. Na identificação de variáveis associadas com anemia, a presença de covariáveis confundidoras foi avaliada por meio de análise de regressão logística multivariada. Foram incluídas no modelo as covariáveis que apresentaram valores de p inferiores a 0,20 na análise bivariada. Considerou-se como estatisticamente significante os valores de p menores que 0,05. As análises foram realizadas com o auxílio do programa *Statistical Package for the Social Sciences*, versão 16.0 para Windows (SPSS Inc., Chicago, IL, EUA).

O estudo foi aprovado no Comitê de Ética em Pesquisa da Faculdade de Medicina do Hospital Universitário Clementino Fraga Filho da Universidade Federal do Rio de Janeiro (Protocolo no. 004/05 em 28/04/2005).

## Resultados

Foram incluídos 166 pacientes, sendo 126 (75,9%) do sexo masculino. A média de idade dos pacientes foi de 39,0 ± 10,7 anos. Em nossa amostra, 62,5% (95/152) dos pacientes eram não-brancos; 18,7% (18/96) estavam infectados por HIV; 64,7% (97/150) foram considerados etilistas através do questionário CAGE; 74,7% (118/158) foram classificados como fumantes e ex-fumantes, e 30,1% (47/156) relataram uso de drogas ilícitas. Entre os 166 pacientes, 18 (10,9%) eram não anêmicos e 148 (89,1%) apresentavam anemia, sendo essa do tipo ferropriva em 4 (2,4%) e do tipo da doença crônica em 126 (75,9%); não foi possível essa diferenciação nos 18 pacientes restantes.

Foram observados baixos níveis de hemoglobina (média, 10,86 ± 2,04 g/dL) em 89,2% dos pacientes; de transferrina (média, 177,28 ± 58,71 mg/dL), em 65,3%; e de VGM (média, 82,00 ± 7,77 fL), em 39,7%; assim como níveis elevados de ferritina (média, 520,68 ± 284,26 ng/mL), em 52,7%; de RDW (média, 16,36 ± 3,47%), em 55,4%; de PCR (média, 5,84 ± 4,22 mg/dL), em 98,2%; e de VHS (média, 60,30 ± 39,84 mm/h), em 84,3%.

Segundo o IMC, a proporção de pacientes com baixo peso foi de 68,7% (n = 88/128; média, 18,21 ± 2,93 kg/m^2)^. Utilizando-se a DCT como parâmetro, observou-se desnutrição (leve, moderada e grave) em 78,7% dos pacientes (n = 126/160; média, 6,16 ± 3,83 mm). Por meio da AMB, a desnutrição nos diferentes graus foi observada em 87,9% dos pacientes (n = 138/157; média, 24,41 ± 9,86 cm^2)^.

Na análise das variáveis sociodemográficas e clínicas entre os pacientes anêmicos e não anêmicos, houve uma associação da presença de anemia com sexo masculino (p = 0,03), assim como uma tendência de associação com pacientes fumantes e ex-fumantes (p = 0,05; Tabelas 1 e 2). Na Tabela 3, são descritas as variáveis nutricionais e laboratoriais entre os pacientes anêmicos e não anêmicos. Foram observadas associações da presença de anemia com as seguintes variáveis: IMC (p = 0,0004), VGM (p = 0,03), ferritina (p = 0,0005), RDW (p = 0,0003) e VHS (p = 0,004). Após a realização da análise multivariada, apenas a variável VHS manteve-se como variável independente.

**Tabela 1:** Distribuição de variáveis sociodemográficas estudadas entre os pacientes anêmicos e não anêmicos.a

Variáveis	Anêmicos	Não anêmicos	OR (IC95%)	p
	(n = 148)	(n = 18)		
Sexo				
Masculino	116 (78,4)	10 (55,6)	2,90 (1,05-7,95)	0,03
Feminino	32 (21,6)	8 (44,4)		
Idade, anos^b^	38,6	37,6		0,71
Tabagismo				
Fumantes	70 (47,3)	9 (50,0)	2,70 (0,98-7,41)	0,05
Ex-fumantes	38 (25,7)	1 (5,6)		
Nunca fumantes	32 (21,6)	8 (44,4)		
NI	8 (5,4)	0 (0,0)		
Uso de drogas ilícitas				
Sim	43 (29,1)	4 (22,2)	1,58 (0,49-5,09)	0,43
Não	95 (64,2)	14 (77,8)		
NI	10 (6,8)	0 (0,0)		

NI : não informado

a Valores expressos em n (%), exceto onde indicado

b Valores expressos em média.

**Tabela 2:** Distribuição das variáveis clínicas estudadas entre os pacientes anêmicos e não anêmicos.a

Variáveis	Anêmicos	Não anêmicos	OR (IC95%)	p
	(n = 148)	(n = 18)		
HIV				
Positivo	17 (11,5)	1 (5,6)	2,79 (0,33-23,1)	0,32
Negativo	67 (45,3)	11 (61,1)		
NI	64 (43,2)	6 (33,3)		
Etilismo				
Sim	90 (60,8)	7 (38,9)	2,28 (0,77-6,70)	0,12
Não	45 (30,4)	8 (44,4)		
NI	13 (8,8)	3 (16,7)		
Nível de transferrina				
Baixo	86 (58,1)	10 (55,6)	1,36 (0,48-3,83)	0,55
Normal	44 (29,7)	7 (38,9)		
NI	18 (12,2)	1 (5,8)		
Nível de ferritina				
Elevado	77 (52)	1 (5,6)	24,6 (3,2-191,2)	0,00005
Normal	49 (33,1)	10 (55,6)		
Baixo	4 (2,7)	7 (38,9)		
NI	18 (12,2)	0 (0,0)		
VGM				
Baixo	63 (42,6)	3 (16,7)	3,70 (1,02-13,35)	0,03
Normal	85 (57,4)	15 (83,3)		
RDW				
Baixo	4 (2,7)	12 (66,7)	0,03 (0,004-0,26)	0,0003
Normal	52 (35,1)	6 (33,3)		
Elevado	92 (62,2)	0 (0,0)		
Nível de PCR				
Normal	2 (1,3)	1 (0,6)	0,23 (0,02-2,72)	0,21
Elevado	145 (98,0)	17 (94,4)		
NI	1 (0,7)	0 (0,0)		
VHS				
Normal	19 (12,8)	7 (38,9)	0,23 (0,07-0,67)	0,004
Elevada	129 (87,2)	11 (61,1)		

NI : não informado

VGM : volume globular médio

RDW : red blood cell distribution width (índice de anisocitose eritrocitária)

PCR : proteína C reativa

a Valores expressos em n (%)

**Tabela 3:** Distribuição das variáveis antropométricas estudadas entre os pacientes anêmicos e não anêmicos.a

Variáveis	Anêmicos	Não anêmicos	OR (IC95%)	p
	(n = 148)	(n = 18)		
IMC				
Baixo peso	82(55,4)	6 (33,3)	5,85 (2,00-17,07)	0,0004
Eutrofia	25 (31,1)	11 (61,1)		
Sobrepeso	3 (2,0)	1 (5,6)		
NI	17(11,5)	0 (0,0)		
Estado nutricional (DCT)				
Desnutrição grave	85 (57,4)	9 (50)	2,24 (0,76-6,57)	0,13
Desnutrição leve/moderada	30 (20,3)	2 (11,1)		
Eutrofia	28 (18,9)	6 (33,3)		
NI	5 (3,4)	1 (5,6)		
Estado nutricional (AMB)				
Desnutrição grave	117 (79,1)	11 (61,1)	2,56 (0,74-8,87)	0,12
Desnutrição leve/moderada	8 (5,4)	2 (11,1)		
Eutrofia	15 (10,1)	4 (22,2)		
NI	8 (5,4)	1 (5,6)		

IMC : índice de massa corpórea

NI : não informado

DCT : dobra cutânea tricipital

AMB : área muscular do braço

a Valores expressos em n (%).

Na Tabela 4, são mostrados os resultados da correlação das variáveis nutricionais e laboratoriais com a presença de anemia. Em relação ao IMC e à DCT, observou-se uma média significativamente menor no grupo de anêmicos que no grupo de não anêmicos. Por outro lado, valores elevados de VHS estiveram associados significativamente ao grupo de pacientes anêmicos (p < 0,001). Entretanto, em relação à medida da AMB e os níveis de transferrina, ferritina e VGM, não foram observadas diferenças significativas entre os grupos de pacientes anêmicos e não anêmicos. 

**Tabela 4:** Correlação das variáveis nutricionais e laboratoriais estudadas nos pacientes anêmicos e não anêmicos.

Variáveis	Anêmicos		Não anêmicos		p*
	n	Média ± dp	n	Média ± dp	
IMC	131	18,047 ± 2,85	17	19,55 ± 3,22	0,044
DCT	143	5,85 ± 3,44	17	8,7 ± 5,72	0,003
AMB	140	24,12 ± 9,95	17	26,88 ± 8,92	0,276
Transferrina	130	175,71 ± 59,64	17	189,29 ± 51,06	0,372
Ferritina	130	534,30 ± 266,98	17	416,54 ± 313,34	0,403
VGM	148	81,83 ± 7,99	18	83,42 ± 5,66	0,415
RDW	148	16,54 ± 3,50	18	14,94 ± 2,92	0,065
PCR	147	6,06 ± 4,23	18	4,04 ± 3,89	0,055
VHS	148	69,49 ± 39,56	18	25,89 ± 21,56	< 0,001

IMC : índice de massa corpórea

DCT : dobra cutânea tricipital

AMB : área muscular do braço

VGM : volume globular médio

RDW : red blood cell distribution width (índice de anisocitose eritrocitária)

PCR : proteína C reativa

* Teste t de Student

## Discussão

No presente estudo, observou-se que a tuberculose pulmonar foi mais comum em adultos jovens, do sexo masculino, etilistas, tabagistas, usuários de drogas ilícitas e infectados pelo HIV; tais características são similares ao relatado em séries que avaliaram esse tipo de pacientes internados em hospitais gerais e de referência para tuberculose em nosso meio.^(^ 17 ^,^ 18 ^)^

No presente estudo, a prevalência de anemia (89,2%) foi superior ao descrito em um estudo na Coreia do Sul (32%)^(^ 6 ^)^ e similar a séries descritas na Indonésia (63%),^(^ 7 ^)^ Tanzânia (96%)^(^ 8 ^)^ e Malawi (88%).^(^ 19 ^)^ Observamos, no presente estudo, uma proporção maior de anemia da doença crônica (75,9%) e menor de anemia ferropriva (2,4%), similar ao descrito em outros estudos,^(^ 6 ^,^ 7 ^)^ mas diferente ao relatado em um estudo.^(^ 8 ^)^ Na análise bivariada, a anemia foi mais comum entre pacientes do sexo masculino, o que difere do descrito na literatura,^(^ 6 ^-^ 8 ^)^ mas tal associação não foi confirmada na análise multivariada; do mesmo modo, não se observou uma associação da presença de anemia com a presença de infecção por HIV, como relatado em outros estudos.^(^ 8 ^,^ 19 ^)^

Na avaliação do estado nutricional utilizando o IMC, o baixo peso foi observado em 68,7% dos pacientes, superior ao descrito em um estudo realizado no Peru (21%)^(^ 20 ^)^ e similar a outros realizados no Malawi^(^ 4 ^,^ 21 ^)^ e na Inglaterra,^(^ 22 ^)^ provavelmente devido ao fato de que, naqueles estudos, foram incluídos apenas pacientes internados com uma elevada frequência de infecção por HIV.

Ao avaliarmos a DCT e a AMB, observou-se a presença de desnutrição em, respectivamente, 78,7% e 87,9% dos pacientes. Resultados similares foram descritos na literatura.^(^ 22 ^)^ Em um estudo caso-controle,^(^ 22 ^)^ foram relatadas reduções na DCT e na AMB de 13% e 20%, respectivamente, enquanto essas reduções foram de 35% e 19% em outro estudo, respectivamente.^(^ 21 ^)^

A quase totalidade dos pacientes incluídos em nosso estudo apresentou níveis elevados de PCR e VHS, resultados similares ao descrito na literatura.^(^ 7 ^,^ 23 ^,^ 24 ^)^ Acreditamos que a PCR e a VHS podem ser úteis como marcadores do efeito da terapia e da involução inflamatória, pois seus níveis decresceram ao longo do tratamento antituberculose e se normalizaram, coincidindo com o final da terapia (dados não mostrados).

As concentrações da maioria das proteínas estão elevadas na tuberculose, com exceção de transferrina e hemoglobina, que estão reduzidas. ^(^ 25 ^)^ Em nosso estudo, observamos concentrações baixas de transferrina e elevadas de ferritina, similar ao descrito por outros autores.^(^ 6 ^,^ 7 ^,^ 25 ^,^ 26 ^)^

Em outras condições que não inflamatórias, a ferritina seria o teste mais sensível para o diagnóstico de deficiência de ferro. No entanto, em casos de tuberculose, a dosagem de ferritina deve ser utilizada com cuidado, pois nessas condições ela não expressa corretamente a quantidade de ferro no organismo. Logo, os pacientes podem apresentar deficiência de ferro mesmo com valores normais ou aumentados de ferritina.^(^ 13 ^)^

Como foi observada microcitose na maioria dos pacientes, o aumento de RDW poderia ser útil para evidenciar a deficiência de ferro,^(^ 26 ^)^ mas seu papel ainda permanece controverso.^(^ 27 ^)^

Na análise entre os grupos de pacientes com e sem anemia em relação a seu estado nutricional, observou-se que, nos pacientes com anemia, a desnutrição foi mais acentuada, com baixas concentrações séricas de transferrina e elevadas de ferritina, como relatado em um estudo.^(^ 7 ^)^ Em relação ao estado inflamatório, na análise multivariada, a VHS mostrou-se mais elevada no grupo com anemia, havendo uma diferença significativa entre os grupos. Um grupo de autores^(^ 27 ^)^ mostrou que a VHS aumenta em resposta à anemia, o que corrobora os resultados encontrados em nossa série. Entretanto, apesar de termos excluído pacientes com história de tuberculose, em insulinoterapia, em diálise peritoneal ou em hemodiálise, assim como em hemotransfundidos nos 3 meses anteriores, as associações de VHS e PCR com a presença de anemia observadas no presente estudo devem ser confirmadas em outras séries com maior casuística, de preferência com uma maior prevalência de anemia ferropriva e sem a presença de comorbidades como HIV, alcoolismo e tabagismo. 

Como não foi possível utilizar todos os parâmetros indicados para o diagnóstico diferencial entre anemia ferropriva e anemia da doença crônica, como a análise do receptor de transferrina ou da medula óssea,^(^ 13 ^)^ observou-se uma baixa frequência de anemia ferropriva isolada pelos critérios adotados, mas estima-se que alguns pacientes com anemia de doença crônica também fossem portadores de anemia ferropriva, como já descrito em um estudo.^(^ 8 ^)^ Nessas situações, a suplementação de ferro poderia não ser benéfica caso fosse prescrita para todos os pacientes. ^(^ 13 ^)^ Em outro estudo,^(^ 7 ^)^ no final do tratamento antituberculose bem sucedido, a anemia foi corrigida sem a suplementação de ferro na maioria dos pacientes.

Em conclusão, a proporção de pacientes com baixo peso e desnutrição, utilizando-se diferentes parâmetros (IMC, AMB e DCT), foi elevada, bem como a ocorrência de anemia da doença crônica. Além disso, os pacientes anêmicos apresentavam um maior grau de desnutrição que os pacientes não anêmicos.
